# Disseminated *Mycobacterium avium*-Intracellulare Complex Infection Presenting With Disseminated Intravascular Coagulation in an AIDS Patient

**DOI:** 10.1177/2324709617740904

**Published:** 2017-11-14

**Authors:** Folusakin Ayoade, James Cotelingam, Andrew Stevenson Joel Chandranesan

**Affiliations:** 1 Louisiana State University Health Sciences Center, Shreveport, LA, USA

**Keywords:** disseminated, *Mycobacterium avium*-intracellulare complex, histoplasmosis, disseminated intravascular coagulation, bone marrow biopsy

## Abstract

Disseminated *Mycobacterium avium*-intracellulare complex (MAC) infection is one of the relatively common opportunistic infections seen in severely immunocompromised AIDS patients. A constellation of clinical, laboratory, and pathological features involving multiple organ systems are often present in disseminated MAC infection but disseminated intravascular coagulation (DIC) has not been previously described in association with this condition. To our knowledge, this is the first reported case of DIC complicating disseminated MAC infection in an AIDS patient. In this article, we present the case of a 33-year-old AIDS patient with high viral load, CD4 lymphocyte count of 1/mm^3^, who presented with nonspecific symptoms, anemia, thrombocytopenia, and increased lactate dehydrogenase, alkaline phosphatase, and ferritin. She also had abnormal coagulation parameters and features compatible with chronic DIC. Bone marrow biopsy assisted in making the correct diagnosis. She also later grew MAC from blood and sputum cultures. There were no other factors identified after a complete workup to explain DIC in this patient. After commencement of appropriate MAC therapy, she initially had a good response with some improvement of her coagulation parameters. Few months later, however, probably attributable to poor medication compliance, her condition deteriorated with development of thromboembolism, full-fledged DIC, sepsis, and an eventual fatal outcome. This case illustrates the importance of including disseminated MAC in the differential diagnosis of DIC in an AIDS patient.

## Introduction

Disseminated *Mycobacterium avium*-intracellulare complex (MAC) infection is a relatively common complication seen in advanced HIV, especially when the CD4 lymphocyte count falls below 50 cells/mm in the absence of appropriate prophylaxis. Incidence rate has been reported to be as high as 20% at 1 year and 43% at 2 years in one study.^1^ With the more widespread use of antiretroviral therapy, however, the incidence has reduced significantly compared to earlier during the AIDS era.

The symptoms of disseminated MAC are often nonspecific and can overlap with other opportunistic infections, including disseminated histoplasmosis.^2^ Disseminated intravascular coagulation (DIC) is a serious complication that may occur in the setting of infection, trauma, or malignancy.^3^ Here, we describe an unusual presentation of disseminated MAC infection associated with DIC in an advanced AIDS patient.

## Case Presentation

A 33-year-old woman with long-standing AIDS, poorly compliant with antiretroviral (ART) treatment, presented with weight loss, progressive weakness, subjective fevers, and a witnessed seizure activity. Her HIV viral load was 989 155 copies/mL and CD4 count was 1/mm^3^. Her ART regimen consisted of emtricitabine/tenofovir disoproxil and dolutegravir. Her past medical history consisted of seborrheic dermatitis and candida esophagitis. She lived in South Central United States and had no significant recent travels. She had one dog but no cats.

Vital signs showed blood pressure of 106/80 mm Hg, pulse 113 beats/min, respiration 19 cycles/min, temperature 99.4°F, and oxygen saturation of 100% on room air. Physical examination was unremarkable except for moderate muscle wasting, moderate ascites, and 3+ pitting pedal swelling.

Laboratory findings showed cytopenias (hemoglobin 5.9g/dL; platelet counts 4100/µL) and white blood cells 9300/µL. D-dimer was elevated (3.6 µG/mL) while serum fibrinogen was low (189 mg/dL). Prothrombin time and partial thromboplastin time were also abnormal at 17.8 seconds and 52.7 seconds, respectively. International normalized ratio was 1.53. Peripheral blood smear showed schistocytes, spherocytes, smudge cells, ovalocytes, and tear drops. Albumin was 1.0 g/dL, aspartate aminotransferase was 92 U/L, and alkaline phosphatase (ALP) was 325 U/L. Alanine aminotransferase, total bilirubin, and kidney function were within normal limits. Procalcitonin and lactic acid were also within normal limits. Lactate dehydrogenase (LDH) was elevated at 469 U/L, as well as serum ferritin at 4770 ng/mL.

Her cerebrospinal fluid chemistry and microbiologic analysis were completely normal. Brain computed tomography scan was negative for hemorrhage or mass. Chest radiograph showed no significant findings, but abdominal ultrasound suggested liver cirrhosis and ascites.

The patient was diagnosed with low-grade DIC. The differential diagnoses at the time of her initial evaluation included disseminated histoplasmosis, mycobacterial infections, cryptococcosis, and less likely viral hepatitis. She was empirically started on intravenous liposomal amphotericin B in addition to intravenous vancomycin and piperacillin/tazobactam.

**Figure 1.**
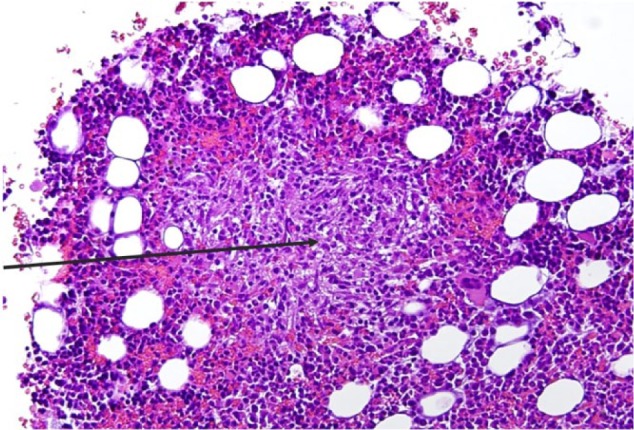
Hematoxylin and Eosin stain of bone marrow aspirate showing a granuloma (black arrow in the center of the granuloma) Magnification x 100.

**Figure 2.**
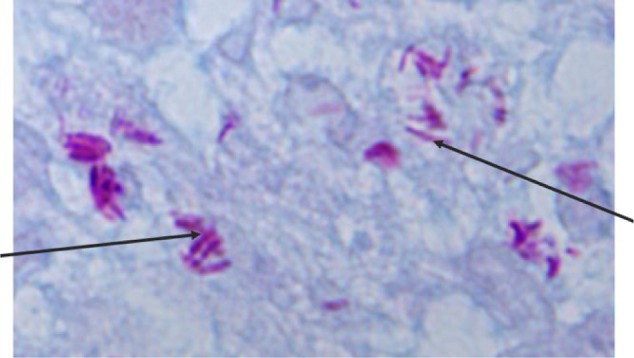
Ziehl-Neelsen stain of bone marrow aspirate showing numerous acid-fast bacilli (black arrows). Magnification x 1000.

Workup for viral hepatitis A, B, and C, histoplasma, cryptococcus, cytomegalovirus, and herpes simplex virus was negative. Blood cultures were obtained for routine bacterial and fungal cultures. Paracentesis was done, with analysis of ascitic fluid showing a transudate and culture negative for fungal and mycobacterial growth. Given her profound cytopenia, bone marrow biopsy was also obtained. Findings on bone marrow touch preparation showed granulomatous inflammation and acid-fast bacilli (Figures 1 and 2). The bone marrow isolate was confirmed by culture as MAC on nucleic acid probe. The same pathogen was later isolated from fungal blood cultures and sputum cultures. Susceptibility testing showed minimum inhibitory concentration (MIC) for clarithromycin = 2, rifampin MIC = 8, and ethambutol MIC = 16.

The patient was started on oral ethambutol 15 mg/kg once daily, rifabutin 300 mg once daily, and clarithromycin 500 mg twice a day. Antifungal therapy and other antibiotics were discontinued. She initially had a favorable response, but 2 to 3 months later, she developed an acute deep vein thrombosis in her right leg and acute pulmonary embolism in the right upper lobe pulmonary branch. She was promptly started on anticoagulation. Even though her platelet counts initially improved, it later plummeted down again to as low as 14/µL with worsening of her coagulation parameters. Four weeks after her pulmonary embolism diagnosis, she developed sepsis and worsening DIC and subsequently succumbed to her infection.

## Discussion

This case illustrates an unusual presentation of disseminated MAC infection. The abnormal coagulation parameters, elevated D-dimer, thrombocytopenia, microangiopathic changes on peripheral blood smear, and venous thromboembolism are more consistent with DIC. There were no other explanations including malignancy present in this patient to account for these abnormal hematological changes.

Our patient with CD4 count of only 1 cell/mm^3^, high viral load, and poor compliance is a perfect host for disseminated MAC infection.

Many patients with disseminated MAC infection have positive blood cultures at the time of diagnosis, with yield ranging from 18% to 37%.^2,4,5^ In a study of HIV patients with CD4 equal to or less than 200 cells/mm^3^, patients who developed MAC infection were more likely to have weight loss, fever, abdominal pain, anemia, elevated LDH, and ALP than matched controls. Several of these symptoms, however, occur a few months before positive blood cultures.^2^

Our case also illustrates the importance of obtaining bone marrow biopsy when the definitive diagnosis is in doubt. Even though the yield of MAC from blood cultures is slightly higher and recovery probably sooner than bone marrow cultures, the finding of acid-fast bacilli from bone marrow aspirate could help in the early initiation of appropriate therapy.^6-8^

Unusual about this case was the DIC, which to our knowledge has not been previously described in association with disseminated MAC in HIV or any other infection. It is unclear if HIV itself might have contributed to DIC in this patient. A few studies have reported impaired coagulation profiles and DIC in advanced HIV (especially with concomitant infectious or neoplastic conditions) compared to those with CD4 >400 mm^3^.^9,10^

DIC has been reported with a few other opportunistic infections in HIV patients including cryptococcosis, histoplasmosis, and pneumocystis.^11-13^ The persistent nature of DIC in this patient and thromboembolism favors chronic DIC over acute DIC even though there is significant overlap. Disseminated MAC, being an infection, may cause DIC as any infection can theoretically cause this unwanted serious condition.^3,14^ The possible mechanism underlying development of DIC in MAC infection is unclear but pathogenesis could be related to elaboration of procoagulants from bacterial products and neutrophils, as suggested for other infectious causes of DIC.^15^ More case reports/series and research are needed to further explore the possible links between disseminated MAC and DIC.

It is long known that disseminated MAC infection can mimic disseminated histoplasmosis and distinguishing between both conditions in patients residing in endemic areas could be sometimes challenging.^16,17^ Factors present in both disease conditions identified in our case include severe immunosuppression, nonspecific symptoms, increased ALP, elevated LDH, elevated aspartate aminotransferase, cytopenias (especially thrombocytopenia), and markedly elevated ferritin. Graviss et al described a model that could be useful in making this distinction.^18^ Even though both conditions are associated with elevated LDH and ALP, according to this model, LDH < 500 U/L and ALP > 300 U/L favors a diagnosis of disseminated MAC infection (our patient had LDH of 469 U/L and ALP of 325 U/L). Also favoring disseminated MAC going by this model is relatively lower CD4 count and relatively higher WBC count, which was also supported by our case.

In conclusion, this case underscores one of the many unfavorable outcomes possible with advanced HIV. Medication compliance was a major challenge with our case and was still a problem even after the patient was started on MAC therapy. It is reasonable to say this probably contributed to her poor outcome.^19^ Disseminated MAC infection should be included in the differential diagnosis of an AIDS patient presenting with acute or chronic DIC.
